# Ataxia, Ophthalmoplegia, and Areflexia: What Would You Think?

**DOI:** 10.1155/2012/150813

**Published:** 2012-06-27

**Authors:** N. Karsan, P. Fletcher, I. Bodi, B. K. MacDonald

**Affiliations:** ^1^Neurology Department, St Georges Healthcare NHS Trust, London SW17 0QT, UK; ^2^Clinical Neuropathology Department, King's College Hospital NHS Foundation Trust, London SE5 9RS, UK

## Abstract

We present here a case of carcinomatous meningitis presenting as Miller Fisher syndrome (MFS). There are four further cases described in the literature with evidence of tumour invasion within the central nervous system (CNS) shown either in cerebrospinal fluid examination or on histology. There are further five cases described in which an association between cancer and a Miller Fisher phenotype has been shown. Some of these have identified antiganglioside antibodies in the serum and, in one case, also showed antibodies deposited within the primary tumour itself. This raises a question as to whether there is a paraneoplastic form. It would be informative when further cases present in this way to histologically examine for malignant CNS invasion, and the presence of antiganglioside antibodies in both the malignant primary and areas of nervous system thought to be affected by MFS.

## 1. Case

A forty-four-year-old man, who was previously fit and well, presented to Accident and Emergency with a three-day history of fever, abdominal pain, headache, and vomiting nine days after returning from a cycling tour in rural India. There was nothing significant to find on examination, and he was afebrile. 

Basic blood tests and cultures showed a mildly raised white cell count and aspartate transaminase (AST) only. An ultrasound scan of the abdomen showed widespread lymphadenopathy in the epigastrium, mesentery, and iliac and para-aortic areas, which was confirmed on CT. Five days after his admission, he developed a complex opthalmoplegia, mild dysphagia, lower limb weakness with areflexia in the upper and lower limbs, and ataxia. There was no sensory involvement, and cognition was normal. Nerve conduction studies showed a motor neuropathy in keeping with the clinical picture of an MFS. Intracranial CT and MRI with contrast were normal, apart from some nonspecific increased signal within the medulla on T2 imaging. Antiganglioside antibodies were negative. A lumbar puncture showed a normal opening pressure and protein, but 35 white cells (90% lymphocytes, 10% polymorphs) and glucose of 2.8 (no matched sample sent) and no organisms on Gram stain. CSF cytology was unremarkable.

Following the ultrasound and CT findings, he was started on antituberculous medication, and following the suspected diagnosis of MFS, he was also started on intravenous immunoglobulin. He went on to have a diverse number of problems which failed to reach a unifying diagnosis. These occurred in quick succession and included

- subclavian vein thrombus,
- sinus tachycardia of 120–140 beats per minute with an ECG showing inferolateral ischaemia. An echo at this time revealed mild inferior wall hypokinesis and a small pericardial effusion,
- peripheral oedema,
- acure renal failure causing ureteric obstruction requiring a nephrostomy,
- respiratory arrest leading to High Dependency Unit admission.

**Figure 1:**
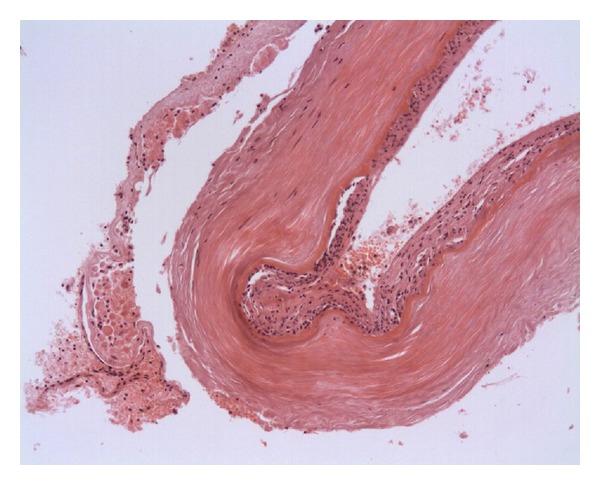
Infiltration of the adventitia and vasa vasorum of the basilar artery by signet ring adenocarcinoma cells.

**Figure 2:**
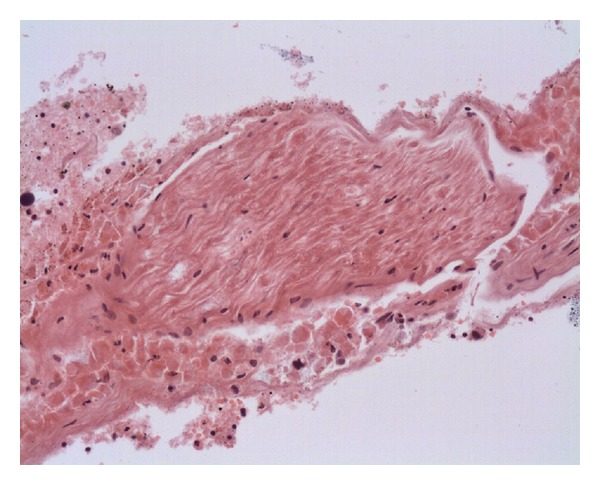
Hypoglossal nerve showing extensive perineural carcinomatous infiltration.

A postmortem examination revealed a poorly differentiated signet cell adenocarcinoma within the pancreas and stomach with diffuse infiltration into the adrenal glands. There was extensive spread within the lymphatic and vascular channels with perineural invasion. The lungs showed multiple small thromboemboli, in keeping with pulmonary microembolisation. A few tumour cells were evident within the thromboemboli. The brain slices showed widespread signet ring carcinoma infiltration into the leptomeninges and brain parenchyma, and in the Virchow-Robin spaces and basilar artery, with occasional small branches of the vertebral artery showing thrombotic occlusion (Figure 1). One sample contained part of the hypoglossal nerve, which showed heavy perineural infiltration (Figure 2).

## 2. Discussion

Secondary invasion of the leptomeninges by systemic cancer was first described by Eberth in 1870 [1], but the term carcinomatous meningitis was not coined until 1912 [2]. Various terms are now used, including neoplastic or metastatic meningitis, to reflect that meningeal invasion can occur secondarily to any form of malignancy. Prior to the advent of CT in the 1970s, myelography was the main imaging modality of choice, and diagnosis was therefore rare. More sophisticated imaging techniques, greater clinical awareness, and the development of better systemically (if not necessarily centrally) acting chemotherapeutic agents, have led to longer life expectancy in systemic malignancies and therefore an increase in the incidence of the condition [3].

Incidence rates vary depending on the case series studied. It is generally accepted that about 5% of patients suffering from metastatic malignancy [4–6] are affected, but estimates are as high as 5–15% for leukaemias and lymphomas, and 1-2% in patients with primary brain tumours [7]. In one postmortem series, patients with known cancer and neurological signs had leptomeningeal involvement in up to 20% of cases [8–10]. The most common type of tumour to metastasise to the leptomeninges is adenocarcinoma or signet ring cell carcinoma as a subtype. In several large series, breast cancer accounted for the majority of cases (11–64%), followed second by lung cancer (14–29%) and third by melanoma (6–18%) [7, 8, 11]. However, these series are dated, and current incidence rates may be different.

Systemic tumour cells have been shown to most commonly enter the CNS via either the arachnoid vessels or choroid plexus following haematological dissemination in both humans [12] and experimental models [13, 14]. Less frequently invasion occurs by infiltration along perineural or perivascular spaces [15–17] or direct spread from parenchymal lesions. The most common sites of deposition are around the skull base, the dorsal surface of the cord, and the cauda EQUINA [7, 18]. It has been postulated that this distribution reflects slower CSF flow around these areas coupled with gravity effects leading to tumour cell deposition. Interestingly, in one study, CSF examination from concomitant ventricular and lumbar sampling showed a discrepancy of positive lumbar, with negative ventricular, cytology in 30% of cases [8].

The clinical presentation can arise from direct cranial or spinal nerve involvement or from complications caused by obstruction of CSF outflow, usually secondary to basal meningitis. In a series of 140 patients [5], the most frequent symptoms were gait problems (46%), headache (38%), altered mental state (25%), and pain and weakness (22%). The most frequent signs were lower motor neuron weakness (78%), absent reflexes (60%), abnormal mental state (50%), extensor plantars (50%), dermatomal sensory loss (50%), ocular paralysis (30%), and facial weakness (25%).

Our patient presented with an ataxia, generalised areflexia, and complex opthalmoplegia mimicking a Miller Fisher syndrome. As can be deducted from the above data, absent reflexes and ocular paresis are fairly common in the cases described, so it may be that his clinical presentation can be fully explained by direct meningeal invasion along the neuroaxis. However, the question arises as to whether this presentation of an exact phenotype of what is thought to be an antibody-mediated syndrome (MFS) suggests a paraneoplastic component to MFS.

Miller Fisher originally described the triad of ataxia, areflexia, and opthalmoplegia in 1956 [19]. The syndrome is thought to be autoimmune in origin, secondary to molecular mimicry between infectious organisms and host gangliosides. For example, with *Campylobacter  jejuni*, a bacterium often implicated, antibodies are found which cross-react with lipopolysaccharides on the bacterial surface and host gangliosides GQ1b/GT1a [20]. Anti-GQ1b gangliosides were first described in Miller Fisher syndrome in 1992 [21], and a strong association was found, with between 83% [22] and 100% (Willison and Veitch, 1994) of cases being positive for GQ1b. However, GQ1b antibodies are not specific to Miller Fisher syndrome, and are found in cases of Bickerstaff's encephalitis (65%) and Guillian-Barré Syndrome (25%), and anti-GQ1b antibodies cross-react with other gangliosides such as GD1b, GT1a, and GD3 in approximately 50% of cases [23]. An association of a predominant oropharyngeal weakness with anti-GT1a seroreactivity, and ophthalmoplegia with anti-GQ1b within the Miller Fisher phenotype, has been described [24–26].

It is unclear whether the pathology of MFS is centrally or peripherally mediated [27], with central nervous system (CNS) involvement clinically (supranuclear palsy and intranuclear opthalmoplegia [28]) and electrophysiologically (abnormal visual, somatosensory, and auditory-evoked potentials) recorded in some cases [29, 30]. The strong association of GQ1b antibodies in Bickerstaff's encephalitis [31, 32], which in addition to a Miller Fisher picture also has superimposed CNS dysfunction, may also add to the idea of both a central and peripheral role to the pathology [33]. High levels of GQ1b have been found both in the nodes of Ranvier [34, 35] and in the nerve trunks [36] of the extraocular cranial nerves (III, IV, and VI) relative to the other cranial or spinal nerves, and this was thought to help explain the ophthalmoplegia seen. However, antiganglioside antibodies have been found to bind to nodes of Ranvier of other nerves and in mouse sciatic nerves [37] (although here this fixation had no effect on the conduction velocities, so the pathological implication of this is not clear), and GQ1b has also been shown to be present at high levels in other unaffected sites. The neuromuscular junction (NMJ) may also be a site of target. In mouse hemidiaphragm preparations treated with anti-GQ1b antibody-positive sera, a temporary increase in acetylcholine release at NMJ is observed [38], and anti-GQ1b antibodies cause structural and functional changes in the NMJ [39]. In humans, abnormalities and electrical “jitter”, a sign of instability of the neuromuscular junction, which improved with clinical recovery, have been demonstrated on electromyography [40].

The anatomical basis of the ataxia is unclear. In Fisher's original paper, he proposed the involvement of 1a afferent fibres, but studies have failed to stain GQ1b in them. Using body sway analysis, Kuwabara et al. [41] have further supported the concept that the ataxia is proprioceptive in origin. Muscle spindle bodies [42] and dorsal roots immunostain for anti-GQ1b antibodies (dorsal roots also stain for GD1b and GD3), but the physiological significance of this is unclear [43]. A study looked at immunostained human cerebellum with anti-GQ1b sera from three Miller Fisher patients [44], and western blot analysis has shown an increase in anticerebellar antibodies in Miller Fisher syndrome patients compared to GBS patients and controls [45]. Therefore, although the site of disease process remains unclear, there is evidence to suggest involvement of peripheral nerve, dorsal root ganglion, neuromuscular junction, and even cerebellum.

In cases such as this where direct evidence of CNS infiltration by malignant cells is seen, it may be that in addition to carcinomatous infiltration of the basal meninges or cauda EQUINA, direct infiltration of areas implicated in the Miller Fisher pathology, such as dorsal root ganglia and/or muscle spindles, leads to the clinical picture of Miller Fisher syndrome. It may well be that a presentation that so distinctly mimicking a syndrome caused by an antibody mediated response is due purely to the chance combination of areas involved. However, as discussed, Miller Fisher syndrome is autoimmune and has also been described in association with autoimmune conditions [46–49], so the exactly same presentation in carcinomatous meningitis raises the question as to whether there is a paraneoplastic component in some cases.

We have found four further cases in the literature where carcinomatous meningitis has presented as Miller Fisher syndrome (Table 1). The first two were described by Guarino et al. in 1995 [50], with the first secondary to a gastric adenocarcinoma and the second to likely myeloma. Both were diagnosed based on positive CSF cytology. Nakatsuji et al. [51] described a case of a woman with a month's history of progressive ataxia, ophthalmoplegia, and areflexia with sensory involvement and sluggish pupillary reflexes on examination. The CSF was lymphocytic, but repeated cytological examination was negative. The MRI showed leptomeningeal enhancement, and a postmortem confirmed widespread leptomeningeal and cranial nerve infiltration by signet ring cell adenocarcinoma. Unfortunately, the family did not permit a postmortem of the rest of the body, so no primary was found. Csépány et al. [52] described a man who presented with a six day history of a progressive right third nerve palsy, bilateral lower motor neurone seventh nerve palsies, hyporeflexia, and ataxia. The CSF cytology showed “signet ring cells” and a few “large atypical cells.” Following this, a chest CT showed a lung mass which on biopsy was revealed to be adenocarcinoma. The CT of his head showed metastatic disease and some enhancement of the right Sylvian fissure. In all four cases, there was clear evidence of active malignancy within the central nervous system, either by CSF examination or by direct evidence of malignant infiltration. However, none of the studies measured anti-GQ1b antibodies, so it is impossible to know whether the clinical picture was accounted for solely by direct malignant infiltration, or whether there was an additional paraneoplastic component.

A literature search looking for an association between malignancy and Miller Fisher syndrome revealed five cases (Table 2) [53–57]. Apart from a small number of white cells (2–14) in the CSF in three cases, none had any direct evidence of malignancy within the CNS on cytology and none had additional information provided by postmortem examination. Anti-GQ1b antibodies were sent in all five cases and were positive in two. It is unclear from these cases which antiganglioside antibodies were tested for. In the cases by Aki et al. and Rubio-Nazabal et al., both patients improved clinically by treatment with plasmapheresis or intravenous immunoglobulin (IVIg), respectively. This may be suggestive of a paraneoplastic mechanism. The GQ1b antibodies were negative in the first case and strongly positive in the second. This patient then went on to have a relapse of Hodgkin's lymphoma, and it would have been interesting to know if the antibody titres normalised with clinical improvement and then increased again with relapse of the malignancy. In our case, antiganglioside antibodies were negative, and there was no response to IVIg.

Interestingly, in the other case with positive anti-GQ1b antibodies (De Toni et al.) [54], as well as high circulating antigangliosides (including GD1a and disialo-GS), high levels of antibodies to the ganglioside GD1 were stained in the primary adenocarcinoma of the lung, with an absence of staining in neoplastic controls (i.e., bronchial adenocarcinoma without a Miller Fisher syndrome), which supports a paraneoplastic cross-reactivity concept.

There is some suggestion that there may be a paraneoplastic component, at least in some cases, which obviously potentially influences management. Therefore, in any future cases, it would be interesting and informative to firstly correlate any antiganglioside activity with time and secondly, in cases unfortunate enough to pass away, to obtain histology from other areas which may be affected by Miller Fisher syndrome, such as the dorsal horns and cerebellum, looking for direct malignant invasion to these areas, and whether these areas and the primary tumours (as in De Toni et al.'s case [54]) stain for antiganglioside antibodies.

## 3. Conclusion

There is therefore some suggestion that there may be a paraneoplastic component, at least in some cases of MFS, which obviously potentially affects management.

## Figures and Tables

**Table 1:** Cases of proven carcinomatous meningitis presenting as Miller Fisher syndrome.

Author	Tumour	Presentation	CSF	NCS	Imaging	Anti-GQ1b	Outcome	Postmortem findings if performed
Guarino et al. [50]	Stomach adeno-carcinoma	Six months after gastrectomy with three days of diplopia, occipital headache. OE: bilateral VI nerve palsies, severe ataxia, and global areflexia	Prot 0.6#x2009;g/L, WCC-1/mL, glu-0.34 g/L. cytology adenocarcinomatous cells	—	CT normal	—	Treated with chemotherapy but worsened and died 20 days later	—
	Myeloma	4 days of diplopia and diffuse arthralgia. Past history of thyroidectomy for cancer 4 years prior and AML 2 years prior. OE: bilateral III nerve palsies, ataxia, and global areflexia	Prot 1 g/L, WCC-290/mL (lymphs), glu-“normal”, Cytology myeloblasts	—	CT normal	—	Treated with intrathecal chemotherapy but died 40 days later	—

Nakatsuji et al. [51]	Signet ring adeno-carcinoma of unknown primary	One month progressive diplopia and unsteadiness. OE: bilateral opthalmoplegia, sluggish pupil light responses, hypo/areflexia, ataxia, and decreased sensation	Opening pressure 12 cm/H20, Prot 2.7 g/L WCC 51 (65% lymphs, 35% polys), Glu-6.4 g/L, Cytology negative	Absent sural/ tibial potentials, and normal motor conduction	MRI with contrast enhancement of III, IV, VI, XII, pons		Rx with IvIg but worsened and died nine weeks after admission	Brain leptomeningeal, cranial nerve, and choroid plexus dissemination of signet right adenocarcinoma

Csépány et al. [52]	Bronchial adeno-carcinoma	Five days of clumsiness of the right arm, double vision, and unsteadiness. OE: right VI nerve palsy, bilateral VII, decreased reflexes, and ataxia	Glu 3.1 g/L, WCC 256 (20% lymphs, 48% macros, 12% monos, 20% large atypical cells, and a few signet ring cells	Mild axonal sensory-motor neuropathy	CT contrast-right sylvian fissure enhancement. MRI few small enhancing cortical regions. CT chest, small lung tumour	—	—	—

**Table 2:** Cases of proven cancer associated with Miller Fisher Syndrome.

Author	Tumour	Presentation	CSF	NCS	Imaging	Anti-GQ1b	Outcome	Postmortem findings if performed
Tahrani et al. [57]	Anaplastic adenocarcinoma of pancreas	Two weeks of worsening mobility and diplopia O/E: bilateral ophthalmoplegia, L LMN VII, areflexia, and generalised weakness	Prot 0.65, WCC 12 (lymphocytes)	—	Non contrast MRI NAD	−ve	Died	Yes but brain not studied

Aki et al. [56]	Chronic lymphocytic leukaemia	Neutropaenia and fever during chemotherapy with diplopia and arm weakness O/E: areflexia, ophthalmoplegia, dysphagia, dysarthria, shoulder weakness, and ataxia	NAD	Axonal sensory motor neuropathy	MRI NAD	−ve	Plasmapheresis with total improvement	—

Gentile et al. [55]	Burkitt's lymphoma	Diplopia O/E: bilateral ophthalmoplegia, areflexia, and gait ataxia	Prot 1.45, no cells, cytology negative	Diffuse axonal neuropathy	MRI NAD	−ve	Some improvement with chemotherapy	—

De Toni et al. [54]	Squamous cell carcinoma of lung	Progressive hand and feet numbness, unsteady gait O/E: general weakness, sensory ataxia, impaired sensation to all modalities, and areflexia	Protein 0.9, WC 14, no cytology	Diffuse axonal sensory polyneuropathy	—	+ve and on lung histology	IVIg, slowly worsened and progressed	—

Rubio-Nazabal et al. [53]	Hodgkin's lymphoma	Fever and weight loss then bilateral diplopia, photophobia, dysphonia, and gait instability on chemotherapy O/E: ophthalmoplegia, fixed and dilated pupils, dysphonia, mild dysphagia, VII palsy, and ataxia	Protein 0.79, WCC 2, normal lymphocytes on cytology	Axonal sensory neuropathy	MRI with contrast NAD	+ve	IVIg, slowly improved over 2 weeks	—
